# A Retrospective, Multicenter Analysis of a Novel Sacroiliac Joint Fusion Device on Safety and Efficacy at 12 Months: Access Study

**DOI:** 10.3390/healthcare13131544

**Published:** 2025-06-28

**Authors:** Michael J. Dorsi, Pankaj Mehta, Chau Vu, Angel Boev, Ashley Bailey-Classen, Greg Moore, David Reece, Alaa Abd-Elsayed, Steven Falowski, Jason E. Pope

**Affiliations:** 1 Department of Neurosurgery, University of California Los Angeles, Westlake Village, CA 91361, USA; michaeldorsi@hotmail.com; 2 Pain Specialists of America, Killeen, TX 78664, USA; mehtapankajmd@gmail.com; 3 Evolve Restorative Center, Santa Rosa, CA 95403, USA; chauvumd@gmail.com; 4 Boev Clinic, Rochester, NY 14621, USA; angelboev@gmail.com; 5 Trinity Pain Medicine Associates, Fort Worth, TX 76104, USA; ashleybaileyclassen@gmail.com; 6 Pacific Sports and Spine, Eugene, OR 97401, USA; gmooremd@mac.com; 7 Absolute Pain Management, Bethesda, MD 20817, USA; docd21@me.com; 8 Department of Anesthesiology, University of Wisconsin, Madison, WI 53707, USA; alaaawny@hotmail.com; 9 Argires-Marotti Neurosurgical Associates of Lancaster, Lancaster, PA 17601, USA; sfalowski@gmail.com

**Keywords:** arthrodesis, sacroiliac joint fusion, sacroiliitis, sacroiliac joint dysfunction, efficacy, safety

## Abstract

**Introduction**: Arthrodesis of the sacroiliac joint (SIJ) has evolved over the last 5 years, with many trajectory strategies emerging. Innovation has outpaced data generation on the safety and efficacy of novel SIJ arthrodesis techniques. This retrospective review of the use of a SiLO TFX SIJ fusion system provides 12-month post-implant outcome data that can be compared with other techniques from published literature. **Methods**: A retrospective analysis was performed on patients that underwent the SiLO TFX sacroiliac joint fusion procedure at eight sites with data on pain reduction and functional improvement from baseline, as measured by a numerical rating scale (NRS) and Oswestry Disability Index (ODI), along with some safety and device integrity assessments recorded at 12 months post-implant. Safety was assessed by identifying key serious adverse events (bleeding, infection, nerve injury), and device integrity was assessed by evaluating misplaced or malfunctioned devices. ODI and NRS outcomes were compared with published rates from the literature. **Results**: Between 16 March 2023 and 20 February 2024, 42 subjects with 12-month ODI data available were enrolled. The subjects had a mean age of 60 ± 11 years, and 71% were female. The mean ODI score of 33 ± 15 at baseline improved to 17 ± 11 at 12 months, with a statistically significant improvement from baseline of 16 ± 15 (*p* < 0.0001). Furthermore, 52% of subjects had a 15-point absolute ODI improvement. Mean NRS of 7.1 ± 2.8 at baseline improved to 2.9 ± 2.2 at 12 months with a statistically significant reduction in pain of 4.2 ± 3.4 (*p* < 0.0001). No key serious adverse events or device integrity complications were noted. Subgroup analyses for a cohort of subjects with baseline ODI ≥ 30 and VAS pain ≥ 50 demonstrated that performance was similar to that in previously published literature with a mean improvement in ODI of 23.3 ± 12.7 (*p* < 0.0001) with 78% of subjects achieving a 15-point improvement at 12 months, and mean NRS improving by 4.7 ± 3.0 (*p* < 0.0001) with 88.9% achieving an improvement of 2 points. **Conclusions**: This data supports the safety and efficacy of SiLO TFX for SIJ fusion. The retrospective outcomes are comparable to those published for lateral-approach SIJ fusion. As follow up is limited to 12 months in this retrospective dataset; long-term fusion and cost-effectiveness remain to be addressed. Prospective, randomized controlled trials with a larger cohort are needed further to compare SiLO TFX to other available SIJ fusion techniques.

## 1. Introduction

Sacroiliac joint disease is becoming a more recognized source for low back pain, with estimates of 15–30% of those patients presenting with the complaint of low back pain [1]. Low back pain (LBP) is a prevalent clinical issue, and the sacroiliac (SI) joint is recognized as a significant source of chronic axial pain. Estimates suggest that the SI joint contributes to pain in approximately one-quarter of patients with chronic LBP [2]. The SI joint functions as a shock absorber, transmitting loads from the spine to the pelvis; however, its complex anatomy and variability (more mobile in women, often stressed by pregnancy/postpartum states) render it susceptible to dysfunction [3]. Patients with SI joint dysfunction (SIJD) typically report unilateral or bilateral low back, buttock, or hip pain that is not midline [3].

Initial management of SIJD is conservative. Core treatments include education, activity modification, and rehabilitative therapy focusing on pelvic girdle stabilization and stretching of the iliopsoas and piriformis muscles. With the market restriction on radiofrequency strategies to manage this disease, treatment options after failure of conservative therapies, including but not limited to physical therapy, bracing, and intraarticular injection, have narrowed to fusion. Spinal–pelvic stability, often under-recognized, plays a central role in sacroiliac joint dysfunction. Loss of joint integrity may result in micromotion and pain, justifying biomechanical fixation strategies such as posterior transfixation.

Recent guidelines have been published for treating SIJ pain [4]. Historically, open SIJ arthrodesis had limited success and high morbidity; however, in the last two decades, minimally invasive (MIS) fusion techniques have proliferated. In a multicenter trial, Polly et al. reported that patients receiving MIS SIJ fusion with triangular implants experienced markedly larger improvements in pain and disability at 2 years compared to those with non-surgical management [5]. A recent systematic review similarly concluded that MIS SIJ fusion (predominantly using the iFuse system) is “probably more effective than conservative management” for chronic SIJD, with large reductions in pain and improvements in function and quality of life [6]. These trials also demonstrated durable results at 1–2 years, with only a small minority of patients requiring revision surgery (2–4% by 2 years).

More recently, posterior and posterolateral SIJ fusion techniques have been developed. Posterior interpositional fusion involves the placement of an implant (or allograft bone) into the dorsal aspect of the SI joint, anchoring it with sacral and iliac screws. For example, the Rialto SI Fusion System (Medtronic, Dublin, Ireland) uses allograft dowels and screws from a posterior approach. At the same time, the Sacrix system places threaded screws through the posterior iliac crest into the sacrum (a “posterior oblique” technique). The early series of these posterior methods report promising outcomes. Moghim et al. found that MIS posterior SIJ fusion with allograft significantly reduced VAS pain scores (from about 8.3 to 2.7) and opioid use over 12 months, with no serious adverse events [7]. Raikar et al. reported on 19 patients treated with the Sacrix posterior-oblique approach: 18 of 19 patients experienced “excellent” pain relief at a median 12-month follow-up, with solid radiographic fusion in all imaged cases and no procedure-related complications [8]. A meta-analysis by Xu et al. pooled outcomes from 48 studies using lateral, posterior, or posterolateral MIS SIJ fusion techniques. All three approaches produced statistically and clinically significant pain reduction at 6 and 12 months, with pooled improvements of similar magnitude regardless of approach [9]. This suggests that posterior methods may offer equivalent pain relief with lower perioperative morbidity.

Thus, the landscape of SIJ fusion has evolved with new implant systems and trajectories. Contemporary devices vary in design; for example, triangular implants are placed laterally, whereas newer posterior transfixing devices (such as the Aurora Spine SiLO TFX system) utilize a hollow conical spacer and transfixing screws to bridge the ilium and sacrum. Biomechanical studies indicate that such posterior interposition devices can preserve more bone, provide a larger fusion surface, and achieve superior reduction of SIJ motion compared to that with traditional posterolateral screws [10].

In this context of expanding SIJ fusion technology, we undertook a multicenter retrospective analysis (the Access Study) of a novel posterior-transfixing SIJ fusion device (SiLO TFX) to evaluate its 12-month safety and efficacy outcomes. These trials also demonstrated durable results at 1–2 years, with only a small minority of patients requiring revision surgery (2–4% by 2 years).

The purpose of this study is to evaluate the safety and efficacy of the SiLO TFX (Aurora Spine & Pain, Carlsbad, CA, USA) for sacroiliac joint fusion for the treatment of symptomatic sacroiliac joint disease.

## 2. Methods

After an IRB exemption was acquired (WCG IRB Work Order # 1-1836874-1), de-identified data from subjects that were consecutively treated and received sacroiliac joint fusion with the SiLO TFX system from January 2023 through February 2024 and had 12-month outcome data available were enrolled. Patient-reported outcome surveys were used to assess function and pain intensity. Function was assessed employing an Oswestry Disability Index (ODI) score with a range of 0–100, and pain intensity was evaluated with a Numerical Rating Scale (NRS) with a range of 0–10. A subset of key serious adverse events (SAEs), classified as infection, nerve injury, or significant bleeding, were identified and evaluated through chart review for symptom presence. Intra-operative fluoroscopy and postoperative imaging were reviewed to assess misplacement and device integrity [11]. Patient demographics were collected, including age and sex. This study represents the first retrospective, multicenter real-world data collection of safety and efficacy at 12 months utilizing an in-line, posterior transfixing bridge with iliac and sacral screws system (SiLO TFX^©^ Aurora Spine, Carlsbad, CA, USA) for the treatment of sacroiliac joint pain.

The SiLO TFX procedure was performed using an in-line posterior approach with a titanium fixation system, as previously defined [11]. Biomechanical data exists to support this technique. Patients’ access to therapy was within the standard practice of the surgeon, following the published conventional diagnosis, medical necessity, and patient candidacy [12].

Data was collected by the research teams of each site, following the study endpoints, demographics, and procedure records, de-identified, and sent to the sponsor for assimilation and analysis. A third party performed the statistical analysis. Statistical analyses were performed using Stata 18 (College Station, TX, USA). The data was summarized using descriptive statistics, including mean, standard deviation, range, and 95% confidence interval for continuous data and frequencies and percentages for categorical data. A paired Student’s *t*-test was used to test if the difference in ODI and NRS at 12 months from the baseline was statistically different from zero using a 2-sided alpha of 0.05 for each assessment. No imputations were performed for missing data.

Baseline characteristics and 12-month performance from 4 key clinical studies were presented for comparison with this retrospective study. As these studies had inclusion criteria of a baseline ODI ≥ 30 and VAS pain ≥ 50, a comparable subgroup with similarly selected subjects with baseline ODI ≥ 30 and NRS ≥ 5 was created for comparisons. The pain criterion of VAS is on a 0–100 range and was appropriately scaled down for the 0–10 range of NRS.

## 3. Results

Forty-two patients were enrolled at five participating sites with ODI data at pre-procedure and 12 months. The majority of patients were female (71.4%), with a mean age of 60 ± 11 years.

Most, 90%, underwent unilateral treatment (Table 1). The mean baseline pain intensity, as measured by NRS, was 7.1 ± 2.8, and the mean level of function, as measured by ODI, demonstrated a baseline mean of 33 ± 15. All procedures were performed on an outpatient basis with a mean operating time of 59.0 + 10.8 min and estimated blood loss of less than 50 mL (Table 2).

The subset of subjects with pre-procedure ODI ≥ 30 and NRS ≥ 5 was generally similar in characteristics, aside from a higher mean ODI of 45 ± 12 and a higher mean NRS of 8.3 ± 1.7, representing a more dysfunctional cohort with more pain.

Mean ODI and NRS both improved significantly (*p* < 0.0001) at 12 months post-implant by 16 ± 15 and 4.2 ± 3.4, respectively (Table 3). The proportion of subjects achieving a minimum clinically significant difference (MCID) of >15 for ODI and >2 for NRS was 52.4% for ODI and 82.4% for NRS (95% CI).

The subgroup cohort demonstrated better performance as expected, with a mean ODI improvement of 23 ± 13 and 84.2% of subjects achieving MCID at 12 months, and a mean NRS improvement of 4.7 ± 3.0 with 88.9% of subjects achieving MCID.

No device-related adverse events or serious adverse events were reported. (Table 4).

**Figure 1:**
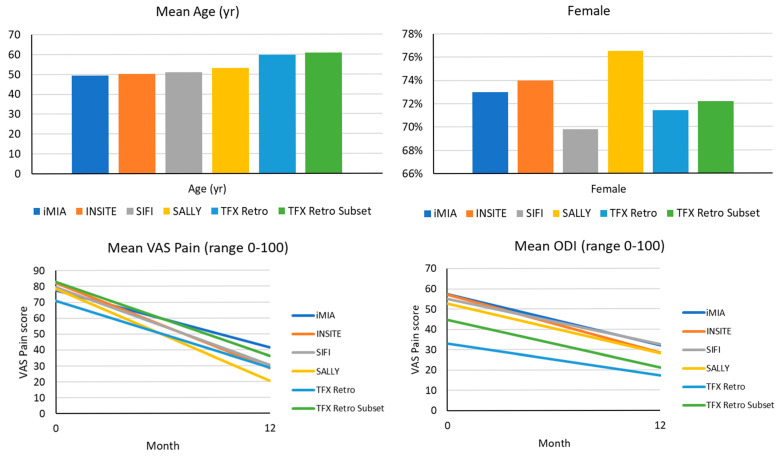
Age, sex, pain, and ODI scores at 12 months, with rates from the published literature.

Published data for the 12-month performance from four clinical trials using the triangular titanium implant (TTI, SI Bone, Santa Clara, CA USA) are presented in Table 5 and Figure 1, along with the whole cohort and subgroup summaries for comparison of cohorts. The SiLO TFX cohort was slightly older (*p* < 0.05) and less impaired via ODI. Still, when restricting the cohort to those with similar baseline ODI and pain assessments, the populations were more comparable concerning ODI and pain levels.

## 4. Discussion

This retrospective study evaluated the 12-month safety and efficacy of SIJ arthrodesis using SiLO TFX, a novel minimally invasive sacroiliac joint fixation device for the treatment of symptomatic sacroiliac joint dysfunction and pain. Patients treated with the SiLO TFX system in our series experienced substantial and sustained reductions in SIJ pain and improvements in function, comparable in magnitude to those reported for other techniques. For instance, our study’s mean VAS pain improvements mirror the large decreases reported in the pivotal randomized trial of triangular titanium implants (mean improvement of ~55 points at 24 months). Similarly, systematic reviews of MIS fusion have consistently found that well-selected patients achieve clinically meaningful pain relief (often ~30–40 mm VAS change) and improved quality of life [13]. Our outcomes at 12 months also appear concordant with the pooled data from diverse fusion methods: Xu et al. found that pain scores improved significantly at 6 and 12 months after lateral, posterior, or posterolateral fusion, with no one approach superior in efficacy [9]. Moghim et al. reported a significant drop in VAS (8.26 to 2.59) and no adverse events at one year for allograft-based posterior fusion [7]; our study observed a similar pattern of rapid pain reduction. In other words, the efficacy and safety profile of the SiLO TFX implant is consistent with the growing evidence that posterior SIJ fusion techniques can deliver durable pain relief with low revision rates. Relative to the published series of posterior fusion, our results also appear favorable. These data represent a clinically meaningful reduction in pain intensity, with 84.2% of the study population achieving a minimal clinically significant difference (MCID) of NRS to be >2 points from baseline, and a mean pain reduction of 4.24 points. Similarly, 52% of patients achieved MCID in functional improvement by ODI score, defined as a 15-point improvement, with a mean decrease of 16 points. The MCID for NRS and ODI was validated by previously published works [14,15]. With the subgroup analysis with a comparable baseline cohort with ODI ≥ 30 and NRS ≥ 5 that many clinical trials require for enrollment into the trial, performance is improved, with mean NRS pain reduced by 4.7, and 88.9% of patients achieving an MCID, and mean ODI improved by 23 points, with 77.8% of patients achieving an MCID. For instance, our cohort’s mean VAS pain improvements mirror the large decreases reported in the pivotal randomized trial of triangular titanium implants (mean improvement ~55 points at 24 months). Similarly, systematic reviews of MIS fusion have consistently found that well-selected patients achieve clinically meaningful pain relief (often ~30–40 mm VAS change) and improved quality of life.

The mean procedure time of 59.0 min is comparable and not statistically significant to the lateral sacroiliac joint fusion, which validates a previous report [16]. In all study participants, no serious adverse events were reported, along with no migration or device malfunction of the implant, similar to a previous analysis.

A unique feature of the SiLO TFX system is its “transfixing” conical spacer combined with ilial and sacral screws. The Neurospine cadaveric study by Raji et al. demonstrated that this posterior interposition technique removed significantly less bone and provided a larger fusion surface area than a single posterior screw [5,13]. Biomechanically, the SiLO TFX construct reduced sacroiliac joint motion by about 42%, substantially more than the 14% reduction achieved by the screw-based posterolateral method, and it maintained the bone-implant interface under fatigue without migration. These findings imply that the device’s design may enhance primary stability and bone ongrowth. In our clinical series, this may translate to the absence of implant-related loosening or migration we observed. The hollow, conical design with multiple lateral windows encourages bony ingrowth, and the posterior placement avoids extensive iliac dissection. To our knowledge, the SiLO TFX is among the first SIJ fusion devices engineered specifically for posterior interpositional fixation, and our results suggest it functions as intended by stabilizing the joint with minimal bone removal.

Despite these encouraging results, the study has several limitations. As a retrospective, nonrandomized analysis, there is inherent selection bias and potential for unmeasured confounding. Patients in this series were treated by a limited number of surgeons with experience in SIJ fusion; our outcomes may not generalize to all practice settings. There was no control group (such as a non-surgical cohort) for direct comparison, so improvements cannot be definitively attributed to the procedure versus placebo or natural history. Blinding was not possible, and patients’ expectations may have influenced subjective outcomes such as pain. The follow-up of 12 months, while standard in SIJ studies, is relatively short; longer-term durability of fusion and symptom relief should be assessed. In addition, radiographic confirmation of fusion was not uniformly available, so we inferred fusion from clinical stability and imaging when done. Opioid reduction was not systematically measured, and our sample size may be insufficient to detect rare adverse events. Finally, our findings come when SIJ fusion technology is evolving rapidly; future developments may further refine the comparative performance of devices.

Future research should address these limitations. Prospective studies—ideally randomized trials—comparing the SiLO TFX system to other established SIJ fusion devices (lateral or allograft) would clarify relative efficacy and safety. Longitudinal studies tracking fusion rates with advanced imaging (e.g., CT) at 2 years and beyond will be essential to verify that the biomechanical advantages translate into solid arthrodesis. Cost-effectiveness analyses and registry data could evaluate the health-economic impact of this new technology. Biomechanically, further cadaver and finite-element studies could explore the effect of implant size and placement on stability. Finally, given the mixed results in the literature (e.g., a recent sham-controlled trial found no significant difference at 6 months [13], it will be critical to continue rigorous trials to substantiate the clinical benefits of SIJ fusion.

In comparison to other published sacroiliac joint surgical treatments, the efficacy of SiLO TFX appears to be comparable [1,6,14,15,16,17,18,19,20,21]. The study cohort in this retrospective study represented a less dysfunctional cohort than the lateral fusion study population in the randomized prospective study cohort, with a baseline mean of 33 versus 57.5, respectively, and a baseline VAS pain intensity of 71 versus 77.1. When specifically comparing the subset of patients with similar baseline dysfunction [*n* = 18], the results were strikingly similar to those seen in the lateral fusion studies.

There are several limitations to this study, including the retrospective nature of the study design, the reliance on a chart dissection for detection of SAEs, the small sample size, the lack of a comparative group, and the lack of radiographic confirmation of fusion with Computed Tomography (CT) [22].

## 5. Conclusions

This data describes the safety and efficacy of SiLO TFX for SIJ fusion, as measured by pain intensity reduction and functional improvement, and supports its use in treating sacroiliac joint dysfunction with a novel in-line transfixation system at 12 months. Further, these outcomes are comparable with published data at 12 months for lateral-approach SIJ fusion. As this is an early, retrospective dataset, long-term fusion and cost-effectiveness will still need to be addressed. Prospective, comparative, randomized controlled trials with a larger cohort are needed to evaluate this novel technique further.

## Figures and Tables

**Table 1:** Characteristics of enrolled subjects.

	Overall (N = 42)	ODI ≥ 30 & NRS ≥ 5 (N = 18)
Age (yr), mean ± SD, (range)	59.7 ± 11.4, (5, 76)	60.9 ± 8.7, (45, 75)
Female, %(*n*)	71.4% (30)	72.2% (13)
Unilateral, %(*n*)	90.5% (38)	94.4% (17)
ODI (0–100), mean ± SD, (range)	33.0 ± 15.2, (9, 70)	44.6 ± 11.9, (30, 70)
NRS pain (0–10), mean ± SD, (range)	7.1 ± 2.8, (0, 10)	8.3 ± 1.7, (5, 10)

**Table 2:** Procedure characteristics.

	Overall (N = 42)	ODI ≥ 30 & NRS ≥ 5 (N = 18)
Operating room time (min), mean ± SD, (range)	59.0 ± 10.8, (44, 91)	57.9 ± 6.9, (45, 69)
Estimated blood loss < 50 mL	100% (42)	100% (18)

**Table 3:** Change in pain intensity reduction as NRS Pain and ODI.

Cohort		N	Baseline	12 Months	Improvement	Achieved MCID *	*p*-Value **
**Overall** **(N = 42)**	ODI (0–100), mean ± SD (range)	42	33.0 ± 15.3 (9, 70)	17.1 ± 10.6 (0, 44)	16.0 ± 14.9 (−12, 50)	52.4% (22)	<0.0001
	(95% CI)		(28.3, 37.8)	(13.7, 20.3)	(11.3, 20.6)		
	NRS Pain (0–10), mean ± SD (range)	38	7.13 ± 2.81 (0, 10)	2.89 ± 2.24 (0, 9)	4.24 ± 3.42 (−7, 10)	82.4% (32)	<0.0001
	(95% CI)		(6.2, 8.1)	(2.2, 3.6)	(3.1, 5.4)		
**ODI ≥ 30 & NRS ≥ 5 (N = 18)**	ODI (0–100), mean ± SD (range)	18	44.6 ± 11.9 (30, 70)	21.2 ± 7.4 (8, 35)	23.3 ± 12.67 (0, 50)	84.2% (32)	<0.0001
	(95% CI)		(38.6, 50.5)	(17.6, 24.9)	(17.0, 29.6)		
	NRS Pain (0–10), mean ± SD (range)	18	8.28 ± 1.74 (5, 10)	3.56 ± 2.59 (0, 9)	4.72 ± 2.95 (0, 10)	88.9% (16)	<0.0001
	(95% CI)		(7.4, 9.1)	(2.3, 4.8)	(3.3, 6.2)		

* Minimal clinically significant difference of >15 points for ODI and >2 points for NRS (equivalent to 20 for VAS). ** Student’s *t*-test, testing for difference from zero.

**Table 4:** Device disruption or displacement and serious adverse events (SAE).

	Procedure	12M
Serious AE (bleeding, infection, nerve injury)	0	0
Device complication	0	0

**Table 5:** Comparisons of SiLO TFX data with the published literature.

Study Name	iMIA (10, 12) NCT01741025	INSITE (1, 13, 14) NCT01681004	SIFI (18) NCT01640353	SALLY (15, 16, 17) NCT03122899	SiLO TFX Retrospective	SiLO TFX Retrospective, Subset ODI ≥ 30, NRS ≥ 5
Design	RCT	RCT	1 arm, prospective	1 arm, prospective	1 arm, retrospective	1 arm, retrospective
Device	TTI	TTI	TTI	3D printed TTI	SiLO TFX	SiLO TFX
**BASELINE CHARACTERISTICS**						
N	52	102	172	51	42	18
Age (yr), mean ± SD (range)	49.4 (27, 70)	50.2 ± 11.4 (25.6, 71.7)	50.9 (23.5, 71.6)	53.2 ± 15	59.7 ± 11.4 (35, 76)	60.9 ± 8.7 (45, 75)
Female	73%	74%	69.8%	76.5%	71.4%	72.2%
Bilateral	32.7%	-	-	9.8%	9.5%	5.6%
**OUTCOMES**						
VAS pain, BL *	77.7 ± 11.3	82.3	79.8 ± 12.8	78.5 ± 11	71 ± 28	83 ± 17
VAS pain, 12M *	41.6 ± 27.0	28.6	30.4 ± 27.6	20.5 **	29 ± 22	36 ± 26
Absolute VAS pain Improvement *	36.1 **	53.7	49.3 ± 29.5	58	42 ± 34	47 ± 29
VAS pain Improved 20 points, 12M	-	81%	-	96%	84.2%	88.9%
ODI, Baseline	57.5 ± 14.4	57.2 ± 13	55.2 ± 11.5	52.8 ± 12.3	33.0 ± 15.2	44.6 ± 11.9
ODI, 12M	32.1 ± 19.9	28.3	31.5 ± 19.2	28	17.1 ± 10.6	21.2 ± 7.4
Absolute ODI improvement	25.4 **	28.9	23.8 ± 20.6	25	16.0 ± 14.9	23.3 ± 12.7
ODI Improved 15 points	-	72.0%	-	67%	52.4%	77.8%

* SiLO TFX collected NRS pain scale of 0–10, scaled up to 0–100 for comparison to VAS of 0–100 as reported in the iMIA, INSITE, SIFI, and SALLY trials. ** Calculated via other data available.

## Data Availability

The data presented in this study are available on request from the corresponding author.
